# Reviving the Lieb–Schultz–Mattis theorem in open quantum systems

**DOI:** 10.1093/nsr/nwae287

**Published:** 2024-08-20

**Authors:** Yi-Neng Zhou, Xingyu Li, Hui Zhai, Chengshu Li, Yingfei Gu

**Affiliations:** Institute for Advanced Study, Tsinghua University, Beijing 100084, China; Institute for Advanced Study, Tsinghua University, Beijing 100084, China; Institute for Advanced Study, Tsinghua University, Beijing 100084, China; Hefei National Laboratory, Hefei 230088, China; Institute for Advanced Study, Tsinghua University, Beijing 100084, China; Institute for Advanced Study, Tsinghua University, Beijing 100084, China

**Keywords:** Lieb–Schultz–Mattis theorem, open quantum system, entanglement Hamiltonian

## Abstract

In closed systems, the celebrated Lieb–Schultz–Mattis (LSM) theorem states that a one-dimensional locally interacting half-integer spin chain with translation and spin rotation symmetries cannot have a non-degenerate gapped ground state. However, the applicability of this theorem is diminished when the system interacts with a bath and loses its energy conservation. In this letter, we propose that the LSM theorem can be revived in the entanglement Hamiltonian when the coupling to the bath renders the system short-range correlated. Specifically, we argue that the entanglement spectrum cannot have a non-degenerate minimum, isolated by a gap from other states. We further support the results with numerical examples where a spin-$1/2$ system is coupled to another spin-$3/2$ chain serving as the bath. Compared with the original LSM theorem that primarily addresses UV–IR correspondence, our findings reveal that the UV data and topological constraints also play a pivotal role in shaping the entanglement in open quantum many-body systems.

## Teaser

The Lieb-Schultz-Mattis theorem, an important result in condensed matter physics that constrains low-energy physics from symmetry and microscopic data, is generalized to open quantum systems.

## INTRODUCTION

Over 60 years ago, Lieb, Schultz and Mattis proved their celebrated result, now known as the Lieb–Schultz–Mattis (LSM) theorem [1,2]. They considered a one-dimensional locally interacting half-integer spin chain with both translation and spin rotation symmetries and showed that this system cannot have a unique ground state with a finite excitation gap. In other words, either the ground states are degenerate, or the low-energy spectrum is gapless. This theorem was later generalized to higher dimensions [3,4] where the ground-state degeneracy can also originate from topological order, bringing out the connection between the LSM theorem and the search for topological spin liquids [5,6]. In the past decades, extensive studies on the LSM theorem have revealed its important role in understanding quantum magnetism and strongly correlated physics [7–30]. Nevertheless, when a quantum system inevitably interacts with the environment, the concept of the energy spectrum is no longer well defined. This raises an interesting issue of whether an LSM-type theorem can still be established for an open quantum system.

In closed systems, the LSM theorem essentially states a deep ultraviolet–infrared (UV–IR) connection. The microscopic details (UV part), such as the spin per unit cell, non-trivially constrain the long wavelength (IR part) property of a many-body spin system. To be concrete, we consider the spin twist operator


(1) \begin{eqnarray*}
\hat{U}^{\text{twist}}=\exp \bigg \lbrace i\frac{2\pi }{L}\sum _{j}j\hat{S}^{z}_{j}\bigg \rbrace ,
\end{eqnarray*}

where *j* labels site index and *L* is the system size. The fact that $\hat{U}^{\text{twist}}$ anti-commutes with the translation operator $\hat{T}$ when acting on a spin-rotationally-invariant state with half-integer spin per unit cell plays a central role in proving the LSM theorem. Note that this anti-commutating relation only relies on the intrinsic feature of the Hilbert space, independent of detailed Hamiltonian forms. Therefore, we anticipate that a certain version of the LSM theorem can also be formulated in open systems.

However, there are multiple choices to replace the role of the energy spectrum when extending the LSM theorem to an open system, without a preferred one *a priori*. For example, Kawabata *et al.* [31] proposed an extension within the Lindbladian framework, which assumes a Markovian bath and constrains the steady-state behavior. In contrast, we provide an alternative proposal without assuming the Markovian bath. When coupled to a bath, the system generically becomes a mixed state, described by a density matrix $\rho$. We write $\rho$ as $\rho =e^{-\hat{K}}$, where $\hat{K}$ is called the entanglement Hamiltonian whose spectrum is called the entanglement spectrum [32]. In this work, we propose that the natural successor of the energy spectrum for an open-system LSM theorem is the entanglement spectrum.

## RESULTS

### Statement and intuition of the open-system LSM theorem

Specifically, we propose an LSM-type theorem for one-dimensional open systems as follows (see the online supplementary material for further discussions of $\mathbb {Z}_2\times \mathbb {Z}_2$ symmetry and spin-1 models): the low-lying spectrum of the entanglement Hamiltonian $\hat{K}$ either has a degenerate minimum or is gapless when the following two conditions are satisfied.

1. *Translation and ${\rm SO}(3)$ symmetries and half-integer representation.* These are key ingredients of the original LSM theorem and can be imposed on an open system. Namely, we consider a half-integer spin chain coupled to a bath (see the online supplementary material). Both the translation and spin rotation symmetries in our setting are the so-called ‘weak symmetries’ for the density matrix $\rho$ of the system [33], i.e. $\hat{U} \rho \hat{U}^\dagger =\rho ,$ or, equivalently,
  (2) \begin{eqnarray*}
\hat{U} \hat{K} \hat{U}^\dagger =\hat{K},
\end{eqnarray*}
  where $\hat{U}$ is either translation or spin rotation acting on the system. When we have specific modeling of the bath, this weak symmetry condition is equivalent to requiring the total state of the full system, including the bath and system-bath coupling, to be invariant under the symmetry. We note that the original LSM theorem can be generalized to the case of discrete symmetries, including $\mathbb {Z}_2\times \mathbb {Z}_2$ (see [8] and the online supplementary material) and time reversal symmetry [11]. These weaker symmetry conditions also suffice in the open system setup. For simplicity and concreteness, we focus on the SO(3) version below.
2. *Short-range correlation.* In contrast to the long-range correlation implied by the closed-system LSM theorem, we assume that the spins in the open system are short-range correlated due to the coupling to the bath, i.e. $\langle \hat{O}_i\hat{O}_j\rangle -\langle \hat{O}_i \rangle \langle \hat{O}_j\rangle \sim e^{-|i-j|/\xi}$ for local operators $\hat{O}_i$ and $\hat{O}_j$ at sites *i* and *j* with a finite correlation length $\xi$. The expectation value $\langle \cdot \rangle$ is taken with respect to the system density matrix $\rho$. We argue that this is the natural result when a system that satisfies the LSM theorem is coupled to a bath. For instance, one scenario is that the system alone is enforced to be gapless by the LSM theorem, while the system-bath coupling is a relevant perturbation and opens up a finite gap $\Delta$. Hence, the system and bath together form a gapped ground state with $\xi \sim 1/\Delta$ roughly the inverse gap. Another scenario is that the system reaches thermal equilibrium with a thermal bath at inverse temperature $\beta =1/T$. In this case, $\xi \sim \beta$ is roughly the thermal length. More generally, because the system-bath coupling is local, quantum spins at different spatial locations alter the environment differently, leading to the loss of long-range coherence.

**Figure 1.**
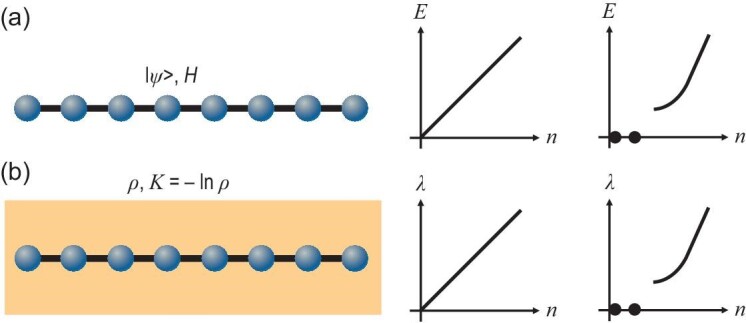
Comparing the LSM theorem in closed and open systems. (a) In closed systems, we consider the physical Hamiltonian ${H}$ and the energy spectrum *E*. (b) In open systems, we consider the entanglement Hamiltonian ${K}$ and the entanglement spectrum $\lambda$. These two spectra exhibit the same features, i.e. either gapless or degenerate ground states, when the LSM theorem holds.

We compare this open-system LSM theorem with that for closed systems in Fig. 1. Given that ‘entanglement’ serves a pivotal role in our proposal, we may alternatively call this open-system LSM theorem the *entanglement LSM theorem*.

Before discussing the details, we first present a special case to gain some physical intuitions into how the open-system entanglement spectrum is related to the energy spectrum. Let us consider two spin-1/2 chains labeled *a* and *b* with the same Hamiltonian $\hat{H}_{a,b}=\hat{H}$ that obeys the conditions for the original LSM theorem, e.g. we assume that $\hat{H}=J\sum _i \hat{\bf S}_{i}\cdot \hat{\bf S}_{i+1}$ is the anti-ferromagnetic Heisenberg model. We then turn on a strong inter-chain anti-ferromagnetic coupling of strength $\Delta$ and treat $\hat{H}_{a,b}$ as perturbations: To the zeroth order, each rung forms a singlet with the singlet–triplet energy gap $\Delta$ and $\Delta \gg J$ is much larger than the typical energy scale of $\hat{H}_{a,b}$. The wave function is a product of spin singlets


(3) \begin{eqnarray*}
\mathinner {|{0}\rangle }=\frac{1}{2^{L/2}}\bigotimes _{i=1}^L (\mathinner {|{\uparrow }\rangle }_{i,a}\mathinner {|{\downarrow }\rangle }_{i,b}-\mathinner {|{\downarrow }\rangle }_{i,a}\mathinner {|{\uparrow }\rangle }_{i,b}),
\end{eqnarray*}

where *i* labels sites. Now, including the first-order change of the wave function induced by the perturbation $\hat{H}_{a,b}$, we have


(4) \begin{eqnarray*}
\mathinner {|{\psi }\rangle } \propto |0\rangle -\frac{1}{2\Delta }(\hat{H}_a+\hat{H}_b)|0\rangle \approx e^{-\beta (\hat{H}_a+\hat{H}_b)/2}|0\rangle
\end{eqnarray*}

with $\beta =1/\Delta$. In the first step, we have simplified first-order perturbation theory using the property that the Heisenberg Hamiltonian $\hat{H}_{a,b}$ flips exactly two singlets to triplets and therefore the energy difference is always $2\Delta$. Furthermore, one can show that the quantum state $\mathinner {|{0}\rangle}$ can be re-written as


(5) \begin{eqnarray*}
\mathinner {|{0}\rangle }=\frac{1}{2^{L/2}}\sum _n\mathinner {|{E_n}\rangle }_a\mathinner {|{\overline{E_n}}\rangle }_b\!,
\end{eqnarray*}

where $\lbrace \mathinner {|{E_n}\rangle }\rbrace$ is the set of complete eigenstates of $\hat{H}$ and the overbar denotes time reversal. With equation (5), it is straightforward to trace out the ‘bath’ *b* in state $|\psi \rangle$ and obtain the reduced density matrix for *a*, which is proportional to $e^{-\beta \hat{H}}$. Therefore, the entanglement spectrum satisfies the LSM theorem.

### Towards an information theoretical proof

Now, we present a few steps that we believe are key to achieving a rigorous proof of the open-system LSM theorem in one dimension.

**Figure 2.**
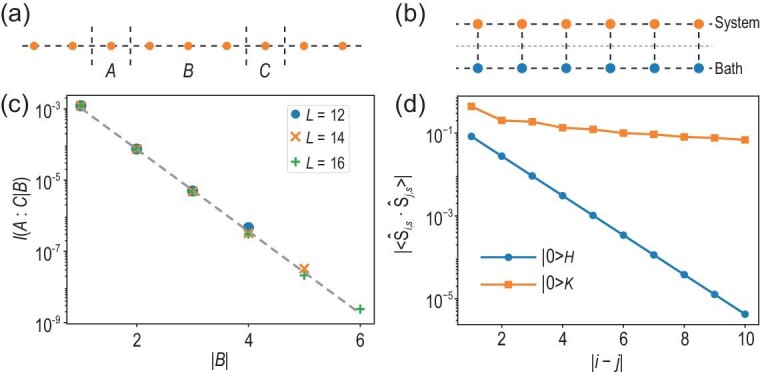
(a) The tripartition *A, B* and *C* of the spin chain as used in the proof. (b) Schematic of the model used in our numerical example. The upper chain with spin-$1/2$ denotes the system and the lower chain with spin-$3/2$ denotes the bath. They are coupled and the entire system respects translation and spin rotation symmetries. (c) The log-linear plot of the conditional mutual information $I(A:C|B)$ (see the text for a definition) as a function of the size of subsystem *B*. (d) The log-linear plot of the spin–spin correlation function $|\langle { \hat{\bf S}}_{i,s}\cdot { \hat{\bf S}}_{j,s}\rangle |$ of system ‘*s*’ as a function of the distance $|i-j|$ under the ground state $|0\rangle _H$ of the physical Hamiltonian $\hat{H}$ (blue circles) and the ground state $|0\rangle _K$ of the entanglement Hamiltonian $\hat{K}$ (orange squares). Note that $|0\rangle _K$ lives in the Hilbert space of system *s*, which is a subspace of total Hilbert space where $|0\rangle _H$ lives. In (d) we have $L=40$.

The first step is to argue that the state in question is a quantum approximate Markov state. This involves the notion of (conditional) mutual information. For two disjoint subsystems *A* and *B*, the mutual information measuring the correlation between *A* and *B* is defined as $I(A:B)\equiv S_A+S_B-S_{AB}$, where *S* is the von Neumann entropy of a subsystem labeled by its subscript. Then, the conditional mutual information between three disjoint subsystems *A, B* and *C* is defined as $I(A:C|B)\equiv I(A:BC)-I(A:B)=S_{AB}+S_{BC}-S_B-S_{ABC}$. The vanishing of the conditional mutual information (i.e. $I(A:C|B)=0$) roughly means that all correlations between *A* and *C* are bridged by *B* and that there is no direct correlation between *A* and *C*. Consequently, a quantum Markov state is defined as the state satisfying $I(A:C|B)=0$. Now, consider the tripartition *A, B* and *C* shown in Fig. 2(a), where *B* shields *A* from *C*. When the total system is short-range correlated, we expect that $I(A:C|B)< \varepsilon$, where $\varepsilon$ is a small parameter controlled by $e^{-l/\xi}$ with *l* the size of subsystem *B* and $\xi$ a correlation length. Such a state is called a quantum $\varepsilon$-approximate Markov state [34–37]. While the exponential decay behavior is beyond rigorous proof so far, it is consistent with our numerics that will be discussed momentarily.

The second step is to show that the entanglement Hamiltonian of a quantum approximate Markov state is local. For a quantum Markov state with $I(A:C|B)=0$, it is known that the entanglement Hamiltonian is strictly local (finite range); more specifically, $\hat{K}_{ABC}=\hat{K}_{AB}+\hat{K}_{BC}-\hat{K}_B$ and therefore the entanglement Hamiltonian $\hat{K}$ for a large patch can be decomposed into a sum of the entanglement Hamiltonian for smaller patches [38]. Now, for a quantum approximate Markov state with $\varepsilon \sim e^{-l/\xi}$, we conjecture that the corresponding entanglement Hamiltonian $\hat{K}$ is exponentially local, namely, the coefficients for non-local terms decay exponentially with the range of the term.

The third step is to show that the spectral gap of $\hat{K}$ vanishes in the $L\rightarrow \infty$ limit. Following the idea of the original LSM theorem and admitting the exponential locality of $\hat{K}$, we can estimate the ‘mismatch energy’ (the energy cost induced by the spin twist) as


(6) \begin{eqnarray*}
\langle \hat{K} \rangle _{\rm twist}\,{-}\, \langle \hat{K} \rangle _0 & {\sim} & L \int _0^\infty\! e^{-l/\xi } \bigg ( 1{-} \cos \bigg ( \frac{2\pi l}{L} \bigg )\bigg ) dl \\
&{\sim} & \frac{4\pi ^2 \xi ^3}{L} - \frac{16 \pi ^4 \xi ^5}{L^3} + \cdots
\end{eqnarray*}

Here the expectation value $\langle \cdot \rangle _0$ is taken on the ground state of $\hat{K}$ and $\langle \hat{K} \rangle _{\rm twist} = \langle \hat{U}^{\rm twist \dagger } \hat{K} \hat{U}^{\rm twist} \rangle _0$ with the twist operator $\hat{U}^{\rm twist}$ defined in equation (1). Since the ground state is orthogonal to the twisted ground state that carries a different momentum as enforced by the non-trivial commutation relation between $\hat{U}^{\rm twist}$ and translation operator $\hat{T}$, the entanglement spectral gap is upper bounded by the above mismatch energy and therefore vanishes at $L\rightarrow \infty$, given $\xi$ a constant independent of *L*.

### Numerical examples

While there remain small gaps in the derivations above from being mathematically rigorous, the results are valid from physical considerations. Instead of delving into the technical aspects of the quantum information theoretical tools, we fill the gaps with numerical evidence, which can illustrate the ideas more concretely. As shown in Fig. 2(b), we let a spin-$1/2$ chain be the system in consideration and another spin-$3/2$ chain be the bath, which is reminiscent of a larger Hilbert space of the bath. The physical Hamiltonian of our first example is written as


(7) \begin{eqnarray*}
\hat{H}\!=\!\sum _{i=1}^L J_1\big (\hat{{\bf S}}_{i}\cdot \hat{{\bf S}}_{i+1}+\frac{1}{3}(\hat{{\bf S}}_{i}\cdot \hat{{\bf S}}_{i+1})^2\big )+J_2 \hat{{\bf S}}_{i,s}\cdot \hat{{\bf S}}_{i,b},
\end{eqnarray*}

where $\hat{{\bf S}}_{i}=\hat{{\bf S}}_{i,s}+\hat{{\bf S}}_{i,b}$ is the sum of spin operators respectively acting on the system and bath, and $J_{1,2}> 0$. Here $\hat{{\bf S}}_{i,s}$ and $\hat{{\bf S}}_{i,b}$ are the spin operators for the system and bath, respectively. At each rung, the $J_2$ term is minimized by forming a spin triplet, rendering the $J_1$ term in the Affleck–Kennedy–Lieb–Tasaki (AKLT) Hamiltonian [39]. (While operator $\hat{{\bf S}}_i$ lives in a larger Hilbert space, different spin sectors decouple and, when focusing on the ground state, they reduce to spin-1 operators.) This Hamiltonian has a gapped excitation spectrum and a unique ground state. We construct the model in this way such that its ground-state wave function can be written explicitly with the matrix product state representation. We let the total system stay in the ground state $|0\rangle _H$ of this Hamiltonian. Knowing the exact many-body wave function, we can obtain the reduced density matrix $\rho = \mathrm{tr}_{b} (|0\rangle \langle 0 |_H )$ of system ‘*s*’, and therefore the entanglement Hamiltonian $\hat{K}=-\ln \rho$, to a fairly large system size.

In Fig. 2(c), we first demonstrate the aforementioned statement on conditional mutual information, which is a crucial step in the locality discussion. We plot $I(A:C|B)$ as a function of the system size of *B* (the tripartition of *A, B* and *C* is shown in Fig. 2(a)), from which we clearly see an exponential decay, as discussed above. The decay length is found to be $\approx 0.38$, which is of the same order as the correlation length of spin operators $\xi \approx 0.91$ on the ground state of $\hat{H}$, as shown in Fig. 2(d) (blue circles). For comparison, we also compute the spin correlation function in the ground state $|0\rangle _K$ of the entanglement Hamiltonian $\hat{K}$, shown with orange squares in Fig. 2(d), which confirms the implications of our entanglement LSM theorem that state $|0\rangle _K$ cannot be short-range correlated.

**Figure 3.**
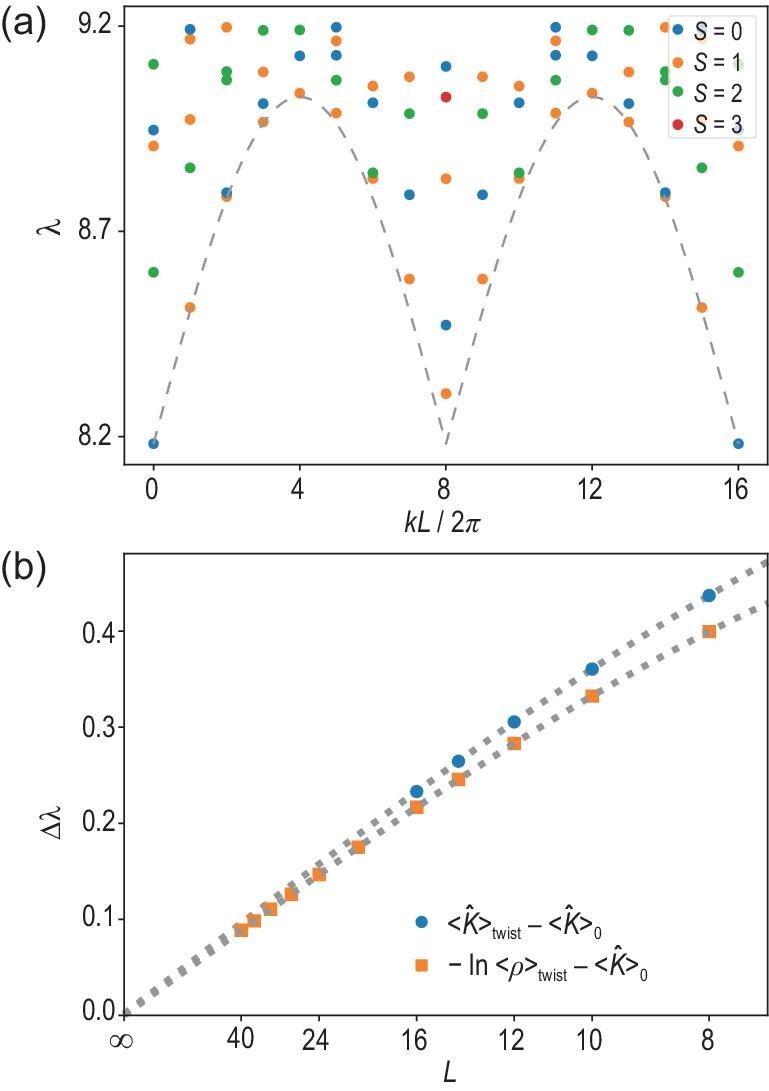
(a) The entanglement spectrum with momentum and total spin resolved. The dashed line is given by $\lambda = v|\sin {k}|+\lambda _0$, with *v* and $\lambda _0$ obtained from the two lowest points at $k=0,2\pi /L$. The calculation is done for $L=16$. (b) Plots of $\langle \hat{K}\rangle _{\text twist}-\langle \hat{K}\rangle _0$ (blue circles) and $-\ln \langle \rho \rangle _{\rm twist}-\langle \hat{K}\rangle _0$ (orange squares) as a function of the total system length *L*. Both quantities are upper bounds of the entanglement spectral gap $\Delta \lambda$ of the entanglement Hamiltonian $\hat{K}$. The dotted lines are fitting curves with $c_0+c_1/L+c_3/L^3$. The $1/L^2$ term and all higher even-order terms vanish due to the inversion symmetry generated by $\exp (i\pi \sum _jS_j^x)$. The fitting results are $(c_0,c_1,c_3)=(0.0014,3.8,-19)$ and $(0.00013,3.6,-23)$ for $\langle \hat{K}\rangle _{\rm twist}-\langle \hat{K}\rangle _0$ and $-\ln \langle \rho \rangle _{\rm twist}-\langle \hat{K}\rangle _0$, respectively. Note that the ratio of coefficients $-c_1/c_3< 3/\pi ^2$ is consistent with the expectation from expanding a ‘mismatch’ energy $\propto 1-\cos ( 2\pi r/L )$, where $r\in \mathbb {Z}$ is the range of the coupling. The ratio reaches the maximum $3/\pi ^2$ when only nearest-neighbor terms are involved.

Next, we calculate the spectral properties of $\hat{K}$ to show the vanishing of the spectral gap in the large system size limit. First, for a total system size $L=16$, we compute the entire entanglement spectrum, as shown in Fig. 3(a). This spectrum is consistent with a linear gapless spectrum; for further numerical discussions and results, see [40]. Then, we focus on the entanglement spectral gap (denoted $\Delta \lambda$) estimation, as discussed above in equation (6). We numerically calculate both $\langle \hat{K}\rangle _\mathrm{twist}$ and $\langle \hat{K}\rangle _0$ and fit the difference against $c_0+c_1/L+c_3/L^3$. A less intuitive but numerically more feasible quantity is $-\ln \langle \rho \rangle _{\rm twist}-\langle \hat{K}\rangle _0$, where $\langle \rho \rangle _{\rm twist}=\langle \hat{U}^{\mathrm{twist}\dagger } \rho \hat{U}^\mathrm{twist}\rangle _0$. Using the convexity of the function $-\ln (x)$, it can be shown that this quantity provides a tighter bound as


(8) \begin{eqnarray*}
\Delta \lambda \leqslant -\ln \langle \rho \rangle _{\rm twist}-\langle \hat{K}\rangle _0 \leqslant \langle \hat{K}\rangle _{\text{twist}}-\langle \hat{K}\rangle _0,
\end{eqnarray*}

where the first inequality originates from the variational principle, a result common to all LSM-related proofs. We demonstrate the numerical results of both quantities in Fig. 3(b). As shown in the data, the finite size scaling supports the statement that the entanglement spectral gap $\Delta \lambda$ vanishes at the thermodynamic limit $L\rightarrow \infty$. Similar numerical results have also been reported previously for two coupled spin-$1/2$ chains [41], which can be regarded as another example supporting our result.

The second model we consider is a spin-1/2 Majumdar–Ghosh (MG) chain [42] coupled to a spin-3/2 bath, where we expect the entanglement Hamiltonian to exhibit spontaneous symmetry breaking. The model reads


(9) \begin{eqnarray*}
\hat{H}&=&\sum _{i=1}^L J_1\bigg (\hat{{\bf S}}_{i,s}\cdot \hat{\bf S}_{i+1,s}+\frac{1}{2}\hat{{\bf S}}_{i,s}\cdot \hat{\bf {S}}_{i+2,s}\bigg ) \\
&&+\, J_2 \hat{{\bf S}}_{i,s}\cdot \hat{{\bf S}}_{i,b}+D(\hat{S}_{i,s}^z+\hat{S}_{i,b}^z)^2.
\end{eqnarray*}

**Figure 4.**
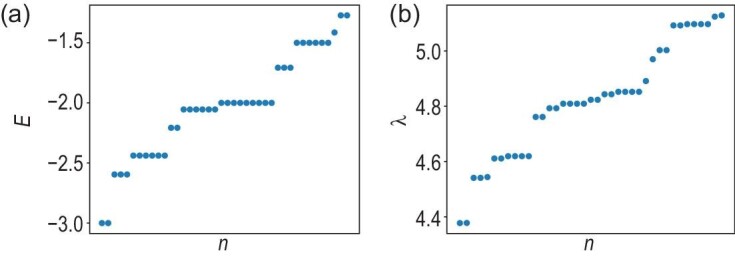
(a) The energy spectrum of the original Majumdar–Ghosh model. (b) The entanglement spectrum of the Majumdar–Ghosh model coupled to a spin-3/2 bath (equation (9)). In both cases, we have the system length $L=8$.

Here we have introduced an anisotropic coupling *D* and set $J_1=J_2=D=1$ in the numerics. Note that we now have $\mathrm{U}(1)\rtimes \mathbb {Z}_2$ instead of SO(3) global symmetry, which also suffices for the LSM theorem [2]. The ground state reduces to a product state of $|S=1, S_z=0\rangle$ in the $J_1\rightarrow 0$ limit, and remains trivially gapped for $J_1=1$, which we have checked numerically. In Fig. 4 we plot the energy spectrum of the original MG model and the entanglement spectrum. In both cases, a doubly degenerate ground-state energy/entanglement eigenvalue characteristic of spontaneous symmetry breaking is observed.

## CONCLUSION AND DISCUSSION

For a spin chain where the original LSM theorem was valid, coupling to the bath overturns the prediction of the LSM theorem. Such coupling can turn the local operators short-range correlated. However, precisely because of the short-ranged correlation, the entanglement Hamiltonian acquires locality, a key ingredient for the revival of the LSM theorem in the entanglement spectrum of an open quantum system. Here we show that familiar topological constraints on the energy spectrum can be extended to the entanglement spectrum of an open system that is entangled with the environment. While compared with existing literature relating the non-trivial topology and the entanglement spectrum, such as the Li–Haldane conjecture [32] where a one-dimensional entanglement cut is performed on a two-dimensional topological state, the entanglement cut discussed in our work is in the same dimension as the system. It will also be interesting to systematically explore the connections between these two scenarios [43–45].

In closed systems, the developments of the LSM-type theorem have revealed its rich content, including the generalization to more sophisticated on-site and space groups [8,9,11,14], symmetry-protected and enriched topological systems [10,12,13,15–19,22–26] and fermionic systems [20,21,28]. These studies have shown an enormous impact on topological and correlated quantum phases. Following the progress made in this work, all these generalizations could also be discussed in the context of the entanglement Hamiltonian for open systems. In parallel to the closed systems, these studies may also strongly impact the understanding of topology and correlation in open quantum many-body systems, an area on the frontier of current theoretical research [46–59] and practically relevant to quantum simulators in the NISQ era [60,61].

Finally, we also note that our discussions on the quantum approximate Markov state and locality of the entanglement Hamiltonian are valid for one dimension, and extending these results into a higher dimension will encounter challenges, such as due to the existence of topologically ordered states. Our open-system LSM theorem predicting a degenerate or gapless entanglement spectrum can manifest itself in a physical quantity such as the full counting statistics [62–65].

## METHODS

In the two numerical examples above, we use a combination of the matrix product state, the density matrix renormalization group (DMRG) and exact diagonalization (ED) methods.

For the AKLT example, the ground state of the ladder $|0\rangle _H$ can be written with a matrix product state exactly. Hence, the reduced density matrix of system $\rho$ can be obtained in terms of a matrix product density operator (MPDO). This form allows us to find the ground state of $\hat{K}$, $|0\rangle _K$, by running the standard DMRG algorithm on $-\rho$, to a very high precision. For our purposes, we find that $L=40$ already suffices, and the ground state $|0\rangle _K$ is used to calculate the correlation function and the twisted state energy, as shown in Figs 2(d) and 3(b).

For the other calculations, DMRG is no longer the best method, either because the ground state of the ladder $|0\rangle _H$ is unknown or because we wish to study further properties of $\hat{K}$ beyond its ground state. In both cases, we restrict to smaller system sizes and use ED. In the AKLT model, we combine the MPDO with ED to calculate the entanglement entropy, which in turn gives the quantum conditional mutual information, as shown in Fig. 2(c). We use ED to obtain the spin- and momentum-resolved low-energy spectrum of $\hat{K}$, as shown in Fig. 3(a). Finally, in the MG example, we use ED to obtain the low-energy spectrum of both $\hat{H}$ and $\hat{K}$, as shown in Fig. 4.

## Supplementary Material

nwae287_Supplemental_Files

## Data Availability

The codes and data for our numerical calculations are available at https://github.com/chengshul/entLSM.
