# Supplementary material for: Reviving the Lieb–Schultz–Mattis theorem in open quantum systems
Source: Natl Sci Rev. 2024 Aug 20;12(1):nwae287. doi: 10.1093/nsr/nwae287 (PMC11719647; doi:10.1093/nsr/nwae287)
Supplement: nwae287_Supplemental_Files [file nwae287_supplemental_files.zip › supp.pdf]

Supplementary Mateirials for "Reviving the Lieb–Schultz–Mattis Theorem in Open Quantum Systems"

Yi-Neng Zhou,[1,*] Xingyu Li,[1,*] Hui Zhai,[1,2] Chengshu Li,[1,†] and Yingfei Gu[1,‡]

[1]*Institute for Advanced Study, Tsinghua University, Beijing 100084, China*
[2]*Hefei National Laboratory, Hefei 230088, China*

(Dated: August 13, 2024)

Further numerical results

To better illustrate the open-system LSM theorem, we present further numerical calculations in this section. We will focus on two cases, one with $\mathbb{Z}_2\times\mathbb{Z}_2$ symmetry and the other with onsite spin-1 Hilbert space. From the discussion in the main text, we expect a degenerate entanglement spectrum in the former case and a generically non-degenerate one in the latter. These are corroborated in the results below.

A model with $\mathbb{Z}_2\times\mathbb{Z}_2$ symmetry.– The symmetry group $\mathbb{Z}_2\times\mathbb{Z}_2$ for spin chains is minimal in the following sense. A large class of systems satisfies the (original) LSM theorem by having both translation symmetry and a projectively realized on-site symmetry. We choose $\mathbb{Z}_2\times\mathbb{Z}_2$ as the onsite symmetry group because it is the simplest one where a projective representation is possible [1, 2]. We consider a spin-1/2 model with the Hamiltonian

$$H=\sum_{i=1}^{L} J_1 S^z_{i,s}S^z_{i,b}+J_2 S^x_{i,s}S^x_{i,b}+J_3(S^z_{i,s}S^z_{i+1,s}+S^z_{i,b}S^z_{i+1,b})+J_4(S^x_{i,s}S^x_{i+1,s}+S^x_{i,b}S^x_{i+1,b}), \tag{S1}$$

which is invariant under π rotations along x and z directions, hence the $\mathbb{Z}_2\times\mathbb{Z}_2$ symmetry. When $J_{1,2}>0$ and $J_{3,4}=0$, the ground state is a product state of spin singlets. The system remains short-range correlated when small $J_{3,4}$ are turned on, and we expect the LSM to hold. A numerical calculation with $J_{1,2}=1, J_3=0.2, J_4=0.1$ is shown in Fig. S1(a), lending full support to the claim.

A spin-1 model.– Now we turn to a spin-1 model where the open system LSM theorem no longer constrains the entanglement spectrum. We consider coupling two AKLT chains,

$$H=\sum_{i=1}^{L} J_1\mathbf{S}_{i,s}\cdot\mathbf{S}_{i,b}+J_2\Big(\mathbf{S}_{i,s}\cdot\mathbf{S}_{i+1,s}+\frac{1}{3}\mathbf{S}_{i,s}\cdot\mathbf{S}_{i+2,s}+\mathbf{S}_{i,b}\cdot\mathbf{S}_{i+1,b}+\frac{1}{3}\mathbf{S}_{i,b}\cdot\mathbf{S}_{i+2,b}\Big), \tag{S2}$$

with $J_{1,2}>0$. The two limiting cases are easy to understand. When $J_2=0$, each rung forms a spin singlet; when $J_1=0$, the two chains are decoupled and each is in the Haldane phase. Crucially, these two limiting cases are in the same phase, with the total system comprising two chains topologically trivial. We thus expect the entanglement spectrum to remain gapped as we tune $J_{1,2}$ interpolating the two limits, which is verified numerically in Fig. S1(b, c), where we take $J_1=1, J_2=0.2$ and $J_1=0.2, J_2=1$ respectively.

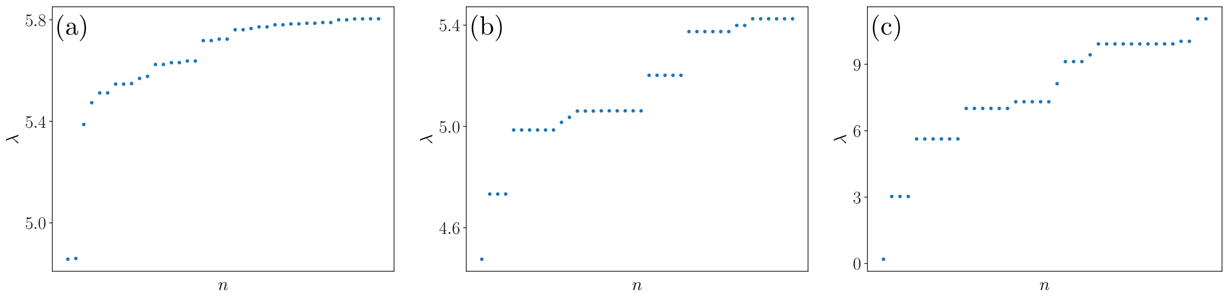

Figure S1. Further numerical results. (a) The entanglement spectrum of a $\mathbb{Z}_2\times\mathbb{Z}_2$ symmetric ladder, where the open system LSM theorem holds and is supported by a two-fold degeneracy. (b, c) The entanglement spectrum of a spin-1 ladder, which receives no constraint from the open system LSM theorem. This is consistent with the non-degenerate entanglement spectrum. We take $L=10$ in (a) and $L=6$ in (b, c).

* These two authors contributed equally.
† lichengshu272@gmail.com
‡ guyingfei@tsinghua.edu.cn

[1] Y. Ogata and H. Tasaki, Lieb–Schultz–Mattis Type Theorems for Quantum Spin Chains Without Continuous Symmetry, Commun. Math. Phys. **372**, 951 (2019).
[2] L. Gioia and C. Wang, Nonzero Momentum Requires Long-Range Entanglement, Phys. Rev. X **12**, 031007 (2022).
