# Supplementary material for: Reviving the Lieb–Schultz–Mattis theorem in open quantum systems
Source: Natl Sci Rev. 2024 Aug 20;12(1):nwae287. doi: 10.1093/nsr/nwae287 (PMC11719647; doi:10.1093/nsr/nwae287)
Supplement: nwae287_Supplemental_Files [file Attachment-3-First-Round-Report.pdf › Attachment-3-First-Round-Report.pdf]

From: **Yingfei Gu** guyingfei@gmail.com
Subject: Fwd: Your_manuscript LK18359 Zhou
Date: January 24, 2024 at 09:22
To: Yi-Neng Zhou zhouyn.physics@gmail.com, Hui Zhai huizhai.physics@gmail.com, Chengshu Li lichengshu272@gmail.com, X li lixy18@mail.ustc.edu.cn

YG

下面是被转发的邮件：

发件人: prl@aps.org
主题: **Your_manuscript LK18359 Zhou**
日期: 2024年1月24日 GMT+8 04:06:14
收件人: guyingfei@tsinghua.edu.cn
回复给: prl@aps.org

Re: LK18359
Lieb-Schultz-Mattis theorem for open quantum systems
by Yi-Neng Zhou, Xingyu Li, Hui Zhai, et al.

Dear Dr. Gu,

The above manuscript has been reviewed by our referees.

The resulting reports include a critique which is sufficiently adverse that we cannot accept your paper on the basis of material now at hand. We append pertinent comments.

Given the conflicting reports, we will likely seek input from additional referees should you resubmit. Because the suitability for Physical Review Letters has been questioned, we request that the revised manuscript makes the impact of the paper clearer.

If you feel that you can convincingly address the concerns, we will give further consideration. With any resubmittal, please include a summary of changes made and a brief response to all recommendations and criticisms.

Yours sincerely,

Martin Rodriguez-Vega, Ph.D. (he/him/his)
Associate Editor
Physical Review Letters
Email: prl@aps.org
https://journals.aps.org/prl/
Follow us on X: @PhysRevLett

NEWS FROM THE PHYSICAL REVIEW JOURNALS

PRL is open to all areas of fundamental or applied physical science
https://go.aps.org/469vJDP
and now publishes forward-looking Essays
https://go.aps.org/essays

Report of Referee A -- LK18359/Zhou

This manuscript discusses the applicability of the Lieb-Schultz-Mattis (LSM) theorem in open quantum systems. The LSM theorem states that a one-dimensional locally interacting half-integer spin chain with translation and spin rotation symmetry cannot have a trivially gapped ground state. The authors generalizes the LSM theorem for the entanglement Hamiltonian of an open quantum system, when the coupling to the bath renders the system short-range correlated. They argue that the entanglement spectrum cannot have a non-degenerate minimum isolated by a gap from other states. The authors support their proposal with numerical examples of a spin-1/2 system coupled to a spin-3/2 chain serving as the bath.

In my opinion, the proof of the generalized LSM theorem for the entanglement Hamiltonian is rather straightforward. In particular, once one admits that the entanglement Hamiltonian K is exponentially local, the logic for proving the LSM theorem in (6) is exactly identical to the well-known original argument by Lieb, Schultz and Mattis, which just utilizes the commutation relation between the twist operator and the Hamiltonian. Accordingly, the spectral consequence for the local entanglement Hamiltonian is exactly the same as the original LSM theorem. This makes me feel that the originality and the level of innovation made in this paper do not reach the high standard of Physical Review Letters.

A relatively non-trivial part of the logic in this manuscript is that they first assume that the system is short-range correlated, and then try to show the exponential locality of the entanglement Hamiltonian based on the assumption. This argument is based on two steps of non-trivial assumptions: 1) the short-range correlated state should be a quantum approximate Markov state, 2) quantum approximate Markov state should have an exponentially local entanglement Hamiltonian.

These two statements are treated as given conjectures in the manuscript and are not considered in detail, and used to rephrase the short-range correlation with the exponentially local entanglement Hamiltonian.

Overall, I am afraid to say that the quality of this manuscript is rather unsatisfying and does not warrant publication in Physical Review Letters. I would suggest the authors to transfer this manuscript to e.g., Physical Review B.

Report of Referee B -- LK18359/Zhou

See Attachment: lk18359_report_1_b.pdf

lk18359_report_1_b.pdf
223 KB

Referee Report for LK18359 Zhou

The paper focuses on a generalization of the established Lieb-Schultz-Mattis (LSM) theorem to the open quantum systems through the entanglement Hamiltonian in an analog to the Hamiltonian in the closed system. Recently, the extensions of LSM theorem beyond pure state have attracted intense interest and the current paper provides another interesting viewpoint from the information theoretical sense. I have several comments and concerns as follows.

1. The original LSM theorem can also cover the discrete symmetries like Z2*Z2 rotation. In my understanding, the results in the current paper do not rely on the SO(3) symmetry quite heavily. Does it mean that the statement can be strengthened similarly?

2. The rigorous proof in the paper demands the condition of one spatial dimension, for example, in my limited understanding, in the locality of quantum entanglement Hamiltonian of a quantum approximate Markov state (Is this true?). The authors are suggested to give more detail on the failure of the proof in higher dimensions.

3. Is there some physical observable related to the LSM theorem of entanglement Hamiltonian? For example, what does a nontrivial degeneracy or gaplessness of the entanglement Hamiltonians, respectively, imply concretely? Several potential applications would be important and helpful for general audience.

The recommendation would be considered after the authors address my comments/concerns above.
