# Supplementary material for: Reviving the Lieb–Schultz–Mattis theorem in open quantum systems
Source: Natl Sci Rev. 2024 Aug 20;12(1):nwae287. doi: 10.1093/nsr/nwae287 (PMC11719647; doi:10.1093/nsr/nwae287)
Supplement: nwae287_Supplemental_Files [file Attachment-4-Second-Round-Report.pdf › Attachment-4-Second-Round-Report.pdf]

From: prl@aps.org
Subject: Your_manuscript LK18359 Zhou
Date: May 2, 2024 at 00:35:29 GMT+8
To: guyingfei@tsinghua.edu.cn
Reply-To: prl@aps.org

Re: LK18359
Lieb-Schultz-Mattis theorem for open quantum systems
by Yi-Neng Zhou, Xingyu Li, Hui Zhai, et al.

Dear Dr. Gu,

This manuscript has been reviewed by our referees and comments from the reports are below.

Based on this, we judge that the work probably warrants publication in some form, but does not meet the Physical Review Letters criteria of impact, innovation, and interest. In accordance with our standard practice, this concludes our review of your manuscript.

The paper, with revision as appropriate, might be suitable for publication in one of the topical Physical Review journals or Physical Review Research. The editors of that journal will make the decision on publication, and may seek further review. However, our complete file is available.

In choosing the journal, please be aware that Physical Review Research is a fully open access publication and, thus, article publication charges would apply.

Please use https://authors.aps.org/Submissions/ to transfer, and include the revised TeX or Word source file, figure files (if revised), a point-by-point response to all recommendations and concerns, and a summary of the changes made.

It might be helpful to also submit an additional PDF file of the manuscript with the revisions highlighted.

Thank you for submitting your work to Physical Review Letters.

Yours sincerely,

Martin Rodriguez-Vega, Ph.D. (he/him/his)
Associate Editor
Physical Review Letters
Email: prl@aps.org
https://journals.aps.org/prl/
Follow us on X: @PhysRevLett

NEWS FROM THE PHYSICAL REVIEW JOURNALS

PRL is open to all areas of fundamental or applied physical science
https://go.aps.org/469vJDP
and now publishes forward-looking Essays
https://go.aps.org/essays

Second Report of Referee A -- LK18359/Zhou

Given that the authors mention the Kawabata-Sohal-Ryu's paper as well as the journal club by Prof. Daniel Arovas, let me start with the comment on these things. First of all, this paper appears is online roughly 5 months later than the work by Kawabata et al., which originally discusses the LSM theorem in the open quantum systems (as far as I can see). While the authors mention that Prof. Daniel Arovas recommends this paper together with the Kawabata et al.'s work on the same ground, I think the fact that Kawabata et al. flew through Physical Review Letters does not necessarily imply that the current work warrants the publication in Physical Review Letters, given the story line of the current work is partly following the story of Kawabata et al.

The authors reply that "We do not refer to the result as rigorous proof simply because it is not hundred percent rigorous in mathematical sense, while the physical considerations already strongly support its validity ". I would say that the step of proving the LSM in the open quantum system is not even in physicist's level of rigor, let alone 100% mathematical rigor. As far as I know, all of the previously known LSM-type theorem in literature is supported by a more or less algebraically rigorous argument involving the algebra of symmetry generators or twist operators. This includes the original theorem for Heisenberg model by LSM, Oshikawa-Hastings for U(1), generalization of LSM to discrete symmetry, crystalline symmetry, higher-form symmetry, SU(N) or other Lie group symmetry, and recent generalization to finite non-invertible symmetry. Also, as far as I can tell, Kawabata et al's proof of LSM is also based on a mathematically rather sound argument — once we define what we mean by gapless or gapped in Lindbladian, the theorem just goes along with the original LSM or Oshikawa-Hasting theorem, and there is no mathematically large gap in there proof. Meanwhile, I feel if I were to use the claimed LSM theorem in the current paper, one still needs to worry if the entanglement Hamiltonian is exponentially local or not (generally, I think there is no guarantee that the modular Hamiltonian created out of a reduced density matrix for local Hamiltonian is also local). Though it is numerically supported by two spin models coupled to a bath, I feel this is still far from established as a stable mathematical truth (even in physicist's sense), and its reliability needs to be distinguished from the many existing generalizations of the LSM theorem in literature.

Given that the title of the paper says "reviving LSM theorem in open quantum systems", I would expect that the central story of the paper is to prove or demonstrate it, at the level of the existing LSM theorem accepted in literature. However, I am afraid to say that the result of the current paper is not stable at that level. Because the central result and the title of the paper is about the *theorem*, I would expect the authors to provide the story about demonstrating or proving the theorem in some minimal level of rigor. Given that the authors say that "A rigorous proof is highly technical and involved, although the physical intuition is quite clear", I assume the authors know how to fill in the gap of the proof, but they are not presenting it simply because it is technical. In order for the current manuscript to get published in Physical Review Letters, I think it is necessary that the authors add Supplemental Material that establishes the proof of the theorem more rigorously. So far, the bulk of the argument is not reliable enough and only the rigorous part of the argument is a straightforward generalization of the original LSM.

In addition, Kawabata et al. have already established that there exists a notion of LSM in open quantum systems. The relatively new perspective of looking at the entanglement Hamiltonian instead of the Lindbladian is nice and I think the new perspective itself is worth publishing somewhere e.g., Physical Review B. However, I am afraid to say that the current new story based on the entanglement Hamiltonian is not wonderful due to the above reasons I mentioned above. I feel this is not enough to say that ‘it substantially advance a particular field’, ‘open a significant new area of research’ compared with Kawabata et al, or satisfyingly ‘solve a critical outstanding problem and therefore pave the way for notable progress in an existing field’.

So far, I am still afraid to say I would like to reject this manuscript.

Second Report of Referee B -- LK18359/Zhou

The current manuscript has addressed my concerns and the publication would be recommended.

** Referee reply to an editorial query after seeing the second report from Referee A.

The current paper under review and the paper by Kawabata et al. are two paralleling works; Zhou et al. is about the mixed state, which is a final equilibrium state, while Kawabata et al. is about the non-equilibrium process without studying the final equilibrium state. Therefore, they are pretty independent works technically and conceptually speaking, although their titles both include “open quantum systems.” Referee A’s first point that the current paper follows Kawabata et al. might be improper.

For the mathematical rigor, Kawabata et al. is not as rigorous as Referee A indicates. Kawabata et al. use a pretty strong assumption on the gap-closing issue, on which we have even no direct physical intuition in a non-Hermitian system. The current paper is physically rigorous, although not 100% mathematical, because it convinces the reader through physical arguments. In this aspect, I am afraid that I could not agree with Referee A on this point, and I think the current paper is mathematically better than Kawabata et al.

Report of Referee C -- LK18359/Zhou

The manuscript proposes a generalization of the LSM theorem for spin-1/2 chains interacting with a bath. For that the authors argue that the LSM theorem can be applied to the entanglement Hamiltonian – given by the density matrix of the system coupled to the bath - when the coupling to the bath keeps the system short-ranged correlated. The authors support their claims numerically by considering spin-1/2 chains coupled to spin-3/2 chains. This is an interesting study but it concentrates only on a limited set of cases.

Some issues are:

1) Throughout the paper, the authors only considered cases, where the system interacting with the bath form paramagnetic states.

2) What happens if the system orders, e.g. if the antiferromagnetic 1D chain is embedded into a 3D Heisenberg antiferromagnet? Also, will a degeneracy of the entanglement spectrum due to spontaneous symmetry breaking of the system interacting with the bath be experimentally detectable? For example, can the "full counting statistics" account for ergodicity breaking?"

3) What happens if the system and bath describe a long-range entangled system, such as a quantum spin liquid, a superconductor or a superfluid in an effective boson-representation for the spin operators? It is not at all evident, why the ground state of \hat{K} will have no long-range entanglement in that case.

4) Some sentences like “Instead of delving into the technical aspects of the quantum information theoretical tools, we fill the gap with numerical evidence” are unfortunate since some more technical details would be useful and some more numerical evidence than the limited cases considered would be welcome.

In summary, the manuscript would gain in impact by including broader and more rigorous validity cases of the LSM theorem in open systems. I would recommend an extension of the manuscript for Physical Review B.

A few minor issues:

- On page 2, the first condition „SO(3) symmetry and half-integer representation“ should also include translation invariance.

- On page 3, the J_1 term in the Hamiltonian (7) is called AKLT Hamiltonian, which is not quite right, as the spin operators in the original paper are spin 1 operators, whereas the operators S_i = S_i,s + S_i,b in Eq. 7 live in a larger Hilbert space. For the sake of clarity, it would be good to point out the difference.

- On page 4 it says „...which confirms the implications of our entanglement LSM theorem that the state l0>_K cannot be short range correlated.“ This may seem a bit confusing to an uncareful reader, as the short range correlation is meant here as a consequence of the LSM theorem on \hat{K}, whereas the state is short range correlated with respect to <S_i, s S_j,s>. Could this description be improved?

- In Figure 2d, the number of sites considered in the calculation is not given.

- In Eq. 8, the second bound, as we understand, is a consequence of Jensen‘s inequality (because ln is a convex function). Could the authors also mention, if the first bound is also a consequence of the convexity of ln, or where does it come from?

FORMS AND MEMOS:

Please see the following:

https://journals.aps.org/prl/authors/resubmittal-policy-physical-review-letters
Resubmittal Policy Physical Review Letters
