# Supplementary material for: Reviving the Lieb–Schultz–Mattis theorem in open quantum systems
Source: Natl Sci Rev. 2024 Aug 20;12(1):nwae287. doi: 10.1093/nsr/nwae287 (PMC11719647; doi:10.1093/nsr/nwae287)
Supplement: nwae287_Supplemental_Files [file Attachment-5-First-Round-Reply.pdf › Attachment-5-First-Round-Reply.pdf]

Dear Dr. Martin Rodriguez-Vega,

First, we would like to thank you and both Referees for carefully reviewing our manuscript.

Referee B has pointed out that extending the LSM theorem to open systems has attracted intense interest. Referee B has made three professional comments on discrete symmetry, extension to higher dimensions, and physical consequences. We have fully responded to these comments, and corresponding changes following each comment have been made in the revised version. We hope Referee B will consider the recommendation after we address these important issues.

Referee A expressed concerns on the level of innovation of our work.

First, Referee A considers the proof of the generalized LSM theorem straightforward. In this reply, we emphasize that the main conceptual challenge of generalizing the LSM to open systems is deciding what quantity we should use to replace the role of the energy spectrum. Our work chooses the entanglement spectrum, and the other paper by Kawabata, Sohal, and Ryu chooses the Lindbladian spectrum, which reveals different aspects of the generalization. The advantage of our choice is that it does not require the Markovian approximation of the bath and can be applied to more general situations. The main technical point is to show that the entanglement Hamiltonian is local when the system-bath coupling renders the correlation short-ranged. Once both the conceptual and technical points are established, the rest of the proof is indeed straightforward. However, it is unfair to ignore these two main contributions and judge this work by the last part.

Secondly, Referee A considers our discussion on entanglement Hamiltonian locality as given conjectures and not being considered in detail. In this reply, we also point out that there was a misunderstanding. We have presented a three-step derivation based on the quantum information theoretical approach. Although there are small gaps in these arguments that

prevent the argument from being one hundred percent rigorous in the mathematical sense, and therefore, we do not refer to our result as rigorous proof, the physical intuitions already strongly support its validity. Moreover, discussing such mathematical details of the proof is not friendly and of broad interest to the general audience, who are unfamiliar with quantum information tools and appreciate physical pictures more than mathematical rigor. Hence, we choose to fill the gap by illustrating numerical examples. We have spent close to two pages on these discussions and numerical results, and we disagree that this part is not considered in detail.

While preparing this reply, a commentary appeared on the Journal Club for Condensed Matter Physics (JCCM), recommending our work in conjunction with the work by Kawabata, Sohal, and Ryu [https://doi.org/10.36471/JCCM_February_2024_01]. This commentary, written by Prof. Daniel Arovas at UCSD, points out that the two recommended works generalize the LSM theorem to open systems with different assumptions and focus on different aspects. At the end of this commentary, Prof. Arovas suggests that it is illustrative to consider a spin-1 chain to contrast with a spin-1/2 chain. It is indeed a good suggestion. We also take this opportunity to add a short session to include a numerical example of the spin-1 chain in the Supplementary Material.

Journal Club for Condensed Matter Physics DOI:10.36471/JCCM_February_2024_01
https://www.condmatjclub.org

Lieb-Schultz-Mattis Theorem for Open Quantum Systems

1. **Lieb-Schultz-Mattis Theorem in Open Quantum Systems**
   Authors: Kohei Kawabata, Ramanjit Sohal, and Shinsei Ryu
   Phys. Rev. Lett. **132**, 070402 (2024) and supplemental material
2. **Reviving the Lieb–Schultz–Mattis Theorem in Open Quantum Systems**
   Authors: Yi-Neng Zhou, Xingyu Li, Hui Zhai, Chengshu Li, and Yingfei Gu
   arXiv:2310.01475

Recommended with a Commentary by Daniel Arovas, University of California, San Diego

We hope you and the referees will find this reply and the revised manuscript have addressed all concerns in the previous report and the revised manuscript is satisfactory for publication in PRL.

Sincerely yours,

Yi-Neng Zhou, Xingyu Li, Hui Zhai, Chengshu Li, and Yingfei Gu

List of changes:

1. On p. 1, we have added a discussion contextualizing our proposal among other approaches to generalizing the LSM theorem to open systems.

2. On p. 1 and p. 2, we have added the qualifier "one-dimensional" in the statement of the open system LSM theorem.

3. On p. 2, we have added a discussion on the generalization to discrete symmetries.

4. On p. 2, we have added further discussion on the condition of short-range correlation.

5. On p. 3, we have added a discussion on the rigorousness of the proof and the rationale of numerical calculations.

6. In the summary, we have added a discussion on the generalization to higher dimensions and referred to some works discussing possible experimental implications of the open system LSM theorem.

7. We have included two numerical calculations illustrating the cases of the spin-1 system and $\mathbb{Z}_2 \times \mathbb{Z}_2$ symmetry in the Supplementary Materials.

8. Some references are updated.

Reply to Referee A:

We thank Referee A for carefully reviewing our manuscript. Below we respond to the comments from Referee A.

This manuscript discusses the applicability of the Lieb-Schultz-Mattis (LSM) theorem in open quantum systems. The LSM theorem states that a one-dimensional locally interacting half-integer spin chain with translation and spin rotation symmetry cannot have a trivially gapped ground state. The authors generalize the LSM theorem for the entanglement Hamiltonian of an open quantum system when the coupling to the bath renders the system short-range correlated. They argue that the entanglement spectrum cannot have a non-degenerate minimum isolated by a gap from other states. The authors support their proposal with numerical examples of a spin-1/2 system coupled to a spin-3/2 chain serving as the bath.

We thank the Referee for a nice summary of our results.

In my opinion, the proof of the generalized LSM theorem for the entanglement Hamiltonian is rather straightforward. In particular, once one admits that the entanglement Hamiltonian K is exponentially local, the logic for proving the LSM theorem in (6) is exactly identical to the well-known original argument by Lieb, Schultz and Mattis, which just utilizes the commutation relation between the twist operator and the Hamiltonian. Accordingly, the spectral consequence for the local entanglement Hamiltonian is exactly the same as the original LSM theorem. This makes me feel that the originality and the level of innovation made in this paper do not reach the high standard of Physical Review Letters.

We thank Referee A for addressing this point, and we would like to take this opportunity

to emphasize our key innovation again.

We think Referee A must agree that there exists a class of important works whose most influential impact comes from pointing out *where to look* rather than solving *how it works*. The LSM theorem is a fundamental theorem for a closed quantum spin system. Generalizing this theorem to an open quantum system is obviously an important issue. However, the most fundamental difficulty is that the statement of the original LSM theorem is made on the energy spectrum, but the energy of an open system is not conserved. Hence, the priority is to identify which quantity we should choose to replace the role of the energy spectrum when generalizing the LSM theorem to an open quantum system.

Here, we should emphasize that there are *multiple choices* to replace the energy spectrum when extending the LSM theorem to an open system. For example, recent research by Kawabata, Sohal, and Ryu [Phys. Rev. Lett. 132, 070402 (2024)] is the only generalization of the LSM theorem to the open system so far. However, they made a completely different choice compared to our work. Their generalization is made within the framework of Lindbladian evolution, and they propose to use the Lindbladian spectrum to replace the energy spectrum. The validity of the Lindbladian framework relies on the assumption of the Born approximation for the coupling between system and environment and the Markovian approximation of the environment. Moreover, the LSM for the Lindbladian spectrum concerns whether there is a gap in the Lindbladian spectrum, which constrains the behavior of a long-time steady state. However, our choice of entanglement spectrum is not subjected to these assumptions of baths and system-bath coupling and is not restricted to the long-time steady state. Instead, we only assume that the coupling to bath renders the correlation short-ranged. The foundation of our work is the density matrix, widely believed to be a universal and natural description of a general open system. The use of the entanglement spectrum as a natural successor to the energy spectrum to generalize the LSM theorem is

the conceptual contribution of this work.

Nevertheless, it is also interesting to see that the LSM theorem generalizations work for both choices of the Lindbladian spectrum and entanglement spectrum, and our work and Kawabata et al.'s work complement each other. In terms of technical challenges, the paper by Kawabata et al. is not more sophisticated than ours. We believe that the reason that the paper by Kawabata et al. is also accepted by PRL is also its physical ideas.

Because of the broad interests of the subject and because our work and Kawabata et al.'s work are complementary to each other, a commentary written by Prof. Daniel Arovas appeared in the Journal Club for Condensed Matter Physics (JCCM) this month [https://doi.org/10.36471/JCCM_February_2024_01], recommending our work together with the work by Kawabata et al. This commentary accurately points out that the two recommended works presume different assumptions, and our approach does not require a phenomenological choice of the jump operators.

Having said that, it is also worth pointing out that the technical point of this work is to show that the entanglement Hamiltonian is local. Referee A also considers this part nontrivial, and we will respond to this point in detail below. In summary, the conceptual point of this work is to identify the entanglement spectrum as the successor of the energy spectrum for the LSM theorem, and the technical point is to show that the entanglement Hamiltonian is local. Once these two points are established, as Referee A said, the rest of the proof of the LSM theorem for open system follows straightforwardly. Nevertheless, despite its straightforwardness, we still need to include this part for the completeness of this work. However, we do not think it is fair to ignore these two most important parts and judge the originality and novelty of our work based on the last and the most straightforward part.

This point might not be emphasized enough in the previous version. In this revision,

we have moved a footnote to the main text and further expanded the discussion into several sentences at the beginning of the third paragraph:

> "However, there are multiple choices to replace the role of energy spectrum when extending the LSM theorem to an open system, without a preferred one a priori. For example, Kawabata, Sohal, and Ryu proposed an extension within the Lindbladian framework, which assumes a Markovian bath and constrains the steady state behavior [31]. In contrast, we will provide an alternative proposal without assuming the Markovian bath."

A relatively non-trivial part of the logic in this manuscript is that they first assume that the system is short-range correlated, and then try to show the exponential locality of the entanglement Hamiltonian based on the assumption. This argument is based on two steps of non-trivial assumptions: 1) the short-range correlated state should be a quantum approximate Markov state, 2) quantum approximate Markov state should have an exponentially local entanglement Hamiltonian. These two statements are treated as given conjectures in the manuscript and are not considered in detail, and used to rephrase the short-range correlation with the exponentially local entanglement Hamiltonian.

Overall, I am afraid to say that the quality of this manuscript is rather unsatisfying and does not warrant publication in Physical Review Letters. I would suggest the authors to transfer this manuscript to e.g., Physical Review B.

We thank Referee A for considering the discussion of the locality of the entanglement Hamiltonian as a nontrivial part. As pointed out above, this is the main technical part of this work. However, we disagree that these two statements are treated as given conjectures in the manuscript and are not considered in detail.

First, we have presented an information theoretical derivation of the locality of the

entanglement Hamiltonian. We do not refer to the result as rigorous proof simply because it is not hundred percent rigorous in mathematical sense, while the physical considerations already strongly support its validity. We believe that this is why Referee B refers to our proof as "rigorous" in the report. We have presented three steps in the derivation, and there are small gaps in the first two steps that prevent the derivation from being completely rigorous mathematically. A rigorous proof is highly technical and involved, although the physical intuition is quite clear. For example, in the current case of quantum approximate Markov chains, Refs. [36–39] attack the issue of localness from various technical aspects, including quantum Gibbs (thermal) states and purely generated finitely correlated states (matrix product states). We do not think it is of broad interest to include such technical and mathematical proof in this work, whose main strength lies in its physical ideas. Instead, we choose to support the claim with numerical simulations. The numerical results agree excellently with our arguments, which are much more friendly for the benefit of a broader audience unfamiliar with the quantum information theoretical tools.

In summary, although there are small gaps from being mathematically rigorous in our three steps of derivation, we have filled the gaps with numerical examples. We believe this is the best way to balance the presentation between being convincing and being of broad interest. We disagree with comments that these statements are not considered in detail, since we have spend two pages on this discussion. To further clarify this point, we have added a few sentence after we present the proof,

> "While there remain small gaps in the derivations above from being mathematically rigorous, the results are valid from physical considerations. Instead of delving into the technical aspects of the quantum information theoretical tools, we fill the gaps with numerical evidence, which can illustrate the ideas more concretely."

Secondly, we would like to emphasize again that our assumption of short-range correlation due to coupling to the environment is a very generic assumption. In the previous version, we presented two generic scenarios in which the coupling to the environment renders the correlation short-ranged. Here we can further present more physical intuitions to strengthen this starting point. The long-range correlation requires quantum coherence between two spatial points separated by a long distance. However, because the coupling between the system and environment is local, quantum spins at different spatial locations change the environment differently. Hence, most naturally, the correlation length is set by the coherent length of the environment, which is generically a finite one. In this revision, we further elaborate this point at the left column of page 2 as follows:

> "More generally, because the system-bath coupling is local, quantum spins at different spatial locations alter the environment differently, leading to the loss of long-range coherence."

With the response and further clarification above, we kindly ask Referee A to reconsider the contributions of our work. We hope Referee A will find this reply and the revision satisfactory for publication in PRL.

Reply to Referee B:

We thank Referee B for carefully reviewing our manuscript. We thank the Referee for insightful and constructive comments, which have greatly helped to improve our manuscript on several important points. Below we respond to the comments from Referee B.

The paper focuses on a generalization of the established Lieb-Schultz-Mattis (LSM) theorem to the open quantum systems through the entanglement Hamiltonian in an analog to the Hamiltonian in the closed system. Recently, the extensions of LSM theorem beyond pure state have attracted intense interest and the current paper provides another interesting viewpoint from the information theoretical sense. I have several comments and concerns as follows.

We thank the Referee for the nice summary of our work.

1. The original LSM theorem can also cover the discrete symmetries like Z2*Z2 rotation. In my understanding, the results in the current paper do not rely on the SO(3) symmetry quite heavily. Does it mean that the statement can be strengthened similarly?

We appreciate the Referee for highlighting the issue of discrete symmetry. It is indeed true that the discrete symmetry generalization of the original LSM theorem applies to our current work. We choose to focus on the SO(3) symmetry version simply because the original LSM theorem was first formulated for SO(3) symmetry, which is more familiar to a broader audience. In our numerical analysis, we have already relaxed the symmetry requirement by considering the $U(1) \rtimes \mathbb{Z}_2$ subgroup in the second example of the Majumdar–Ghosh model (Eq. 9). The Referee's inquiry prompts us to reconsider the necessity of explicitly stating the strongest version of the statement. We have now incorporated a comment on this point. When discussing the statement of the open system LSM theorem, we add

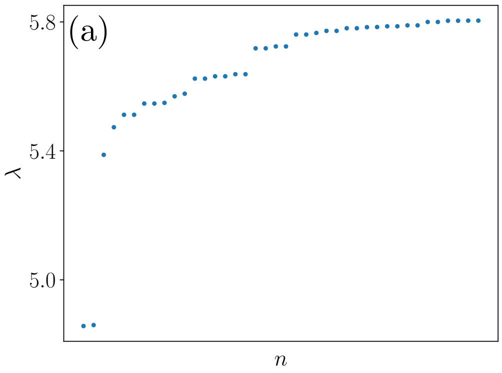

Figure 1: The entanglement spectrum of a $\mathbb{Z}_2 \times \mathbb{Z}_2$ symmetric ladder, where the open system LSM theorem holds and is supported by a two-fold degeneracy. We take $L = 10$ in the calculation.

> "We note that the original LSM theorem can be generalized to the case of discrete symmetries, including $\mathbb{Z}_2 \times \mathbb{Z}_2$ [8,33] and time reversal symmetry [11]. These weaker symmetry conditions also suffice in the open system setup. For simplicity and concreteness we focus on the SO(3) version below."

We also add a numerical calculation of a $\mathbb{Z}_2\times\mathbb{Z}_2$-symmetric model for illustrative purposes in the Supplementary Materials. In the Supplementary Materials, we consider the model

$$H = \sum_{i=1}^{L} J_1 S^z_{i,s} S^z_{i,b} + J_2 S^x_{i,s} S^x_{i,b} + J_3 (S^z_{i,s} S^z_{i+1,s} + S^z_{i,b} S^z_{i+1,b}) + J_4 (S^x_{i,s} S^x_{i+1,s} + S^x_{i,b} S^x_{i+1,b}), \quad (1)$$

which is invariant under π rotations along x and z directions, hence the $\mathbb{Z}_2 \times \mathbb{Z}_2$ symmetry. When $J_{1,2} > 0$ and $J_{3,4} = 0$, the ground state is a product state of spin singlets. The system remains short-range correlated when small $J_{3,4}$ are turned on, and we expect the LSM to hold. A numerical calculation with $J_{1,2} = 1, J_3 = 0.2, J_4 = 0.1$ is shown in Fig. 1, whose results full support the claim.

2. The rigorous proof in the paper demands the condition of one spatial dimension, for example, in my limited understanding, in the locality of quantum entanglement Hamilto-

nian of a quantum approximate Markov state (Is this true?). The authors are suggested to give more detail on the failure of the proof in higher dimensions.

We appreciate the Referee for highlighting the issue about higher dimensions generalization. We believe that this is a very interesting direction warranting further research. It is already a nontrivial work in close systems when the LSM theorem is generalized from one dimension to a higher dimension. For example, in two dimensions, there is an exceptional case compared to one dimension, which is the topologically ordered state. We will encounter similar challenges in open systems when generalizing the LSM theorem to a higher dimension. As Referee B pointed out correctly, the quantum information theoretical tools we used in proving the LSM theorem in 1D do not apply straightforwardly to higher dimensions. For example, there is no proof in higher dimensions that a quantum approximate Markov chain has a local approximate entanglement Hamiltonian. Hence, our current study focuses on one-dimensional systems, and the generalization to higher dimensions demands more studies.

Regarding this comment, we have made the following changes in this revision. First, we have added "one-dimensional" when we first state our LSM theorem in the first page and we state the proof in the second page. Secondly, we added an explicit comment on this point at the end of this paper

> "Finally, we also note that our discussions on quantum approximate Markov state and locality of the entanglement Hamiltonian are valid for one-dimension, and extending these results into a higher dimension will encounter challenges, such as due to the existence of topologically ordered states. "

3. Is there some physical observable related to the LSM theorem of entanglement Hamiltonian? For example, what does a nontrivial degeneracy or gaplessness of the entanglement Hamiltonians, respectively, imply concretely? Several potential applications would be important and helpful for general audience.

The Referee has again raised a very relevant and insightful question that merits and anticipates several publications. Since we completed our work, several related works have been posted on arXiv discussing the experimental consequences of nontrivial degeneracy and the gaplessness entanglement spectrum.

One of our co-authors recently posted a paper (https://arxiv.org/abs/2312.11188) that precisely solves the problem asked by the Referee. The main finding of this paper is to identify an experimental method to observe the distinctive features associated with the open system LSM theorem. Specifically, the paper shows that the nontrivial degeneracy or gaplessness manifests as a singularity in the experimental measurable "disorder operator" defined as

$$f(\theta) := -\lim_{L\to\infty} \frac{1}{L} \ln \left| \langle \mathrm{e}^{\mathrm{i}\theta S^z_{\mathrm{tot}}} \rangle \right| . \tag{2}$$

Similar results have also been discussed in another recent paper by another of our co-authors (https://arxiv.org/abs/2402.15964). Although this paper focuses on the Gaussian state, it also addresses the physical consequence of degenerate or gaplessness entanglement spectrum in the full counting statistics, which can also be applied to the case discussed in this work. At the same period of time, there are also other two papers discussing the related issue (https://arxiv.org/abs/2312.09074, https://arxiv.org/abs/2401.09548). As one can see, this is indeed an important issue that motivates follow-up works.

Following the Referee's suggestion, we have now referred to these works discussing possible experimental implications of the open system LSM theorem at the end of this paper

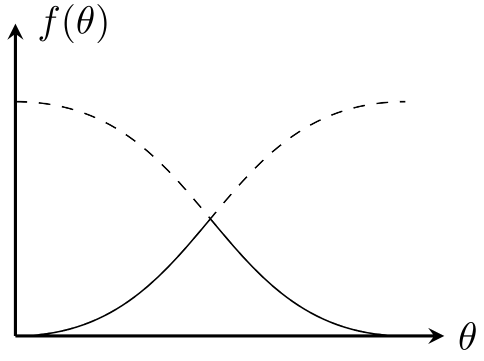

Figure 2: The experimental measurable $f(\theta)$ defined in [arXiv:2312.11188]. As explained in the cited paper, the function has a singularity in the domain $\theta \in (0, 2\pi)$ for an open quantum system with degenerate or gapless entanglement spectrum. The figure is adapted from the preprint.

> "Our open system LSM theorem predicting degenerate or gapless entanglement spectrum can manifest itself in physical quantity such as the full counting statistics [65–68]."

The recommendation would be considered after the authors address my comments/concerns above.

We have fully responded to these three comments made by Referee B and made corresponding changes. We hope Referee B will find this reply and the revision satisfactory.
