# Supplementary material for: Reviving the Lieb–Schultz–Mattis theorem in open quantum systems
Source: Natl Sci Rev. 2024 Aug 20;12(1):nwae287. doi: 10.1093/nsr/nwae287 (PMC11719647; doi:10.1093/nsr/nwae287)
Supplement: nwae287_Supplemental_Files [file Attrachment-6-Second-Reply-Drafted.pdf › Attrachment-6-Second-Reply-Drafted.pdf]

(* This is the draft version of the reply to the second round of referee reports. However, we finally decided not to resubmit this paper back to PRL.)

Dear Dr. Rodriguez-Vega,

We are deeply grateful for your time and effort in reviewing our manuscript and sending us the Referee's report.

Referee B has recommended our paper for publication in PRL and fairly compared our results with those of Kawabata et al.'s paper. Referee C commented that our work only concentrates on limited cases. The main reasons are listed as four issues. However, they are essentially two points: i) We only focus on the paramagnetic states and do not consider symmetry-breaking cases, and ii) we do not consider long-range entangled states such as quantum spin liquid.

However, we have discussed the symmetry-breaking case in our manuscript (see the discussion of the Majumdar-Ghosh model), which is overlooked by Referee C. Moreover, there is no spin liquid state in one dimension.

Referee C considers our work a follow-up to Kawabata et al.'s paper. We strongly disagree with this statement. As we emphasized in the previous reply, these two papers take very different approaches to generalizing the LSM theorem. Their paper focuses on the Lindbladian spectrum, and our work focuses on the entanglement spectrum. The studies of the LSM theorem in closed systems have attracted considerable attention for decades, and its generalization to open systems is undoubtedly a topic that deserves multiple publications in PRL. Referee C also considers our work less rigorous than Kawabata et al.'s paper. We also strongly disagree with this statement. As Referee B pointed out, Kawabata et al.'s paper also made a strong assumption about the gap in the Lindbliadian spectrum. The non-rigorous part of our work is about the locality of entanglement Hamiltonian, a long-standing problem known in the quantum information community. Suppose we follow the same stratagem as Kawabata et al.'s paper to assume the exponential locality of the entanglement Hamiltonian as a prior. In that case, the rest of the proof can follow as rigorously as the LSM theorem proved in the closed system mentioned by Referee A.

We understand that the PRL review process is normally restricted to two rounds. However, since Referee C's report contains obvious misunderstandings of our work, and Referee A's assessment of our work in comparison with Kawabata et al.'s paper is completely unacceptable, we hope that our work can be considered for further review to maintain the justice of the review process. If the review process has been concluded in this round, we would like to apply for a formal appeal in that case. Thank you very much for your consideration.

Best Regards,

The Authors
